# Neutralizing-antibody-mediated protection of chickens against infectious bursal disease via one-time vaccination with inactivated recombinant *Lactococcus lactis* expressing a fusion protein constructed from the RCK protein of *Salmonella enterica* and VP2 of infectious bursal disease virus

**DOI:** 10.1186/s12934-019-1061-9

**Published:** 2019-01-31

**Authors:** Wenqian Wang, Yuxin Song, Linlin Liu, Yuan Zhang, Tingting Wang, Wang Zhang, Kai Li, Xiaole Qi, Yulong Gao, Li Gao, Changjun Liu, Yanping Zhang, Yongqiang Wang, Qing Pan, Gaoming He, Xiaomei Wang, Hongyu Cui

**Affiliations:** 1 State Key Laboratory of Veterinary Biotechnology, Harbin Veterinary Research Institute, Chinese Academy of Agricultural Sciences, Harbin, 150069 China; 2 College of Animal Science and Technology, Shihezi University, Shihezi, 832003 Xinjiang China

**Keywords:** Recombinant lactic acid bacteria, IBDV, RCK, VP2

## Abstract

**Background:**

Infectious bursal disease (IBD) is an acute contagious immunosuppressive disease which lead to acute bursal injury and immune dysfunction in poultry. It has caused heavy economic losses in the commercial poultry industry for many years in worldwide. Attenuated live vaccine has widely used in poultry showing some promising signs against IBDV infection. But it has defects such as generating enhanced virulence and immunosuppression prohibits. Therefore, the development of mucosal vaccines using the food-grade lactic acid bacterium is necessary. Here, we construct a recombinant *Lactococcus* co-expressing the major IBDV antigens VP2 and RCK protein of *Salmonella enterica* to prevent IBD.

**Results:**

The recombinant fusion protein VP2-RCK was expressed in a soluble and stable form in the cytoplasm of the recombinant *Lactococcus lactis*. Animal experiments showed that: (1) the survival rates of the injected immunization inactivated recombinant LAB group and oral immunization live recombinant LAB group were 100% and 80%, respectively; (2) ELISA titers of all serum samples from all experimental groups were negative, but high amounts of specific neutralizing antibodies were detected (1:2^10^ to 1:2^12^); and (3) the bursas of the injected immunization inactivated recombinant LAB group did not suffer damage, as confirmed by clinical observation and bursal histopathological examination. Our results indicate that r-*L. lactis*-OptiVP2-RCK induces a specific neutralizing-antibody-mediated immune response that confers full protection against very-virulent IBDV (vvIBDV) challenge.

**Conclusion:**

*Lactococcus lactis* NZ3900 strain and its matching plasmid pNZ8149 could express the recombinant fusion protein VP2-RCK in a soluble form in the cytoplasm. The protective efficacy of r-*L. lactis*-OptiVP2-RCK (100%) was better than r-*L. lactis*-OptiVP2 (0%) which prove RCK protein played its unique role. The neutralizing antibodies titers against infectious bursal disease virus via one-time vaccination with inactivated r-*L. lactis*-OptiVP2-RCK could reach 1:2^10^ to 1:2^12^, but ELISA titers of all serum samples were negative. For this phenomenon, perhaps because of the change of delivery pathway or the spatial structure of fusion protein. We need further study to test these hypotheses.

## Background

Infectious bursal disease (IBD) is an acute contagious immunosuppressive disease in chickens caused by infectious bursal disease virus (IBDV) [1, 2]. In recent years, the variant IBDV infection has caused serious economic losses and heavily impacted the poultry industry [3–5]. The chicken is most susceptible to IBDV infection at 3 to 6 weeks of age, IBDV infects these young chickens through the digestive tract and massively destroys B cells in the bursa of Fabricius (BF, a primary lymphoid organ), and it is the time at the maximal stage of BF development, and then the consequent immunosuppression increases susceptibility to other infectious diseases and the risk of subsequent vaccination failure as well [6–9]. Although a weak and attenuated live vaccine has shown some promising signs against IBDV infection in poultry, IBD live (attenuated or medium virulent) vaccine strains are effective and widely used, the risk of generating enhanced virulence and immunosuppression prohibit the use of these vaccines in most situations [9, 10], immunization of these strains results in bursa damage and produces immunosuppression, which lead to an impaired immune response to other vaccinations. Moreover, with the high levels of circulating maternal antibodies, the immunity of attenuated live vaccines of IBDV can be easily inhibited [9, 11–13]. Therefore, there is a clear need to develop a new vaccine strategy in poultry. VP2 protein is the major host-protective antigen found in IBDV structural protein of capsid. It encompasses different independent epitopes responsible for the induction of neutralizing antibodies that passively protect chickens and is the major protective antigen of IBDV [7, 13–15]. Therefore, VP2 was expressed as a target antigen protein in many studies [14, 16–18]. And our previous study shows that the live (not inactivated) recombinant *L. lactis* VP2-OmpH strain is a promising candidate vaccine to prevent IBDV infection [18].

Lactic acid bacteria (LAB) are a type of Gram-positive bacteria that produce lactic acid through carbohydrate fermentation. Most LAB species benefit animals, plants and humans. For thousands of years, LAB have been widely and successfully applied for food fermentation [19–22]. Studies investigating the molecular genetics of LAB have revealed that these bacteria also demonstrate promise as live vectors expressing heterologous antigens. LAB live carrier vaccines have broad application potential, particularly as mucosal live vaccine carriers [19, 20, 23, 24]. LAB expression systems are far less common than *E. coli* expression systems. Usually, they are not as efficient as *E. coli* systems for the expression of exogenous proteins [20, 21, 25]. In addition, effective and efficient antigen delivery is a key determinant of successful mucosal immunization. The direct expression of exogenous antigen does not induce a satisfactory immune response [16, 26]. Therefore, effective antigen delivery such as antigen internalization APC (antigen presenting cell) cells is crucial to avoid mucosal immunity failure and poor immune performance.

The *rck* gene (Resistance to complement killing, RCK) encodes a 17 kDa outer membrane protein that is homologous to a family of virulence-associated outer membrane proteins including pagC and Ail, RCK protein is associated with a failure to form fully polymerized tubular membrane attack complexes [27, 28]. Previous study showed that Salmonella enterica bacterium could invade and internalize the cells via the RCK outer membrane protein. RCK was necessary and sufficient to enable non-invasive *E. coli* and RCK-coated beads to adhere, and invade different cells through both Zipper and Trigger internalization mechanisms [29]. Previous Rosselin Manon’s research has shown that RCK conferred recombinant *E. coli*-RCK strain with the ability to bind to and invade epithelial (MA104 and HT29), fibroblastic (NIH-3T3), trophoblastic, and endothelial (Jeg-3 and HBrMEC) cell lines, with the highest invasion level obtained in MA104 cells. However, just as importantly, only a 46 amino acid region (residues 113–159) of RCK is essential for cellular binding and internalization [29]. Therefore, “antigen-RCK46 fusion protein” with only essential 46 amino acid region may possibly improve antigen internalization APCs (antigen presenting cells) cells in specific immune response. Following this strategy, in this study we first report the expression of the major IBDV antigens VP2 and RCK fusion protein in *Lactococcus lactis*. VP2-RCK protein were targeted to the cytoplasm, the recombinant *Lactococcus* were used for oral or injected immunization of chickens, and the immune response and neutralizing-antibody were monitored. This is the first report of a trial that used VP2-RCK fusion antigens producing LAB (the LAB was inactivated) in chickens.

## Results

### Construction of recombinant plasmids expressing the VP2-RCK fusion protein in *L. lactis*

**Fig. 1:**
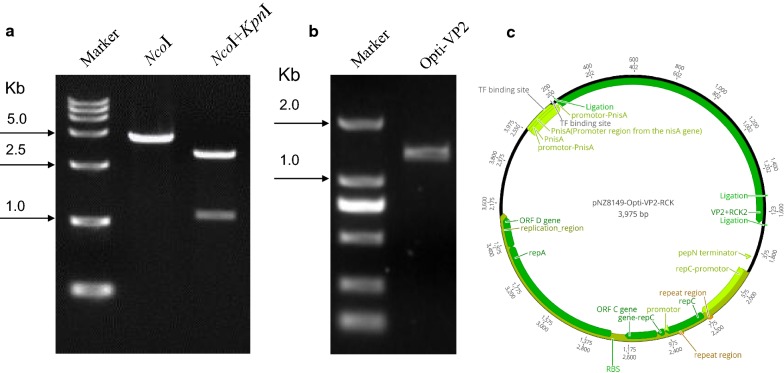
Construction of the plasmid pNZ8149-OptiVP2-RCK expressing the VP2-RCK fusion protein. **a** The recombination plasmid pNZ8149-OptiVP2-RCK was linearized with *Nco*I to obtain a 3975-bp fragment (lane 2). The recombinant plasmid pNZ8149-OptiVP2-RCK was identified via digestion with *Nco*I and *Kpn*I to obtain a 2646-bp fragment and a 1329-bp fragment (lane 3). The molecular mass standard sizes of the DNA marker are indicated to the left. **b** PCR amplification of Opti-VP2 optimization gene (1329 bp). **c** schematic diagrams of the recombinant plasmid pNZ8149-OptiVP2-RCK with the VP2-RCK gene fusion

We designed the VP2-RCK fusion gene to be expressed under the inducible promotor NisA. Briefly, the opti-*rck* gene was inserted into the plasmid pNZ8149 to produce the plasmid pNZ8149-RCK. Opti-VP2 was also amplified (Fig. 1b) and then inserted into pNZ8149-RCK to obtain recombinant pNZ8149-OptiVP2-RCK, which was linearized with *Nco*I to produce a 3975-bp fragment (Fig. 1a, Lane 2). This fragment was digested with *Nco*I and *Kpn*I, resulting in a 1329 bp fragment and a 2646 bp fragment (Fig. 1a, Lane 3). The recombinant plasmid pNZ8149-OptiVP2-RCK was electro-transformed into *L. lactis* NZ3900 to produce r-*L. lactis*-OptiVP2-RCK, which harbored pNZ8149-OptiVP2-RCK (Fig. 1c).

### Recombinant VP2-RCK fusion protein expression in r-*L. lactis* NZ3900

**Fig. 2:**
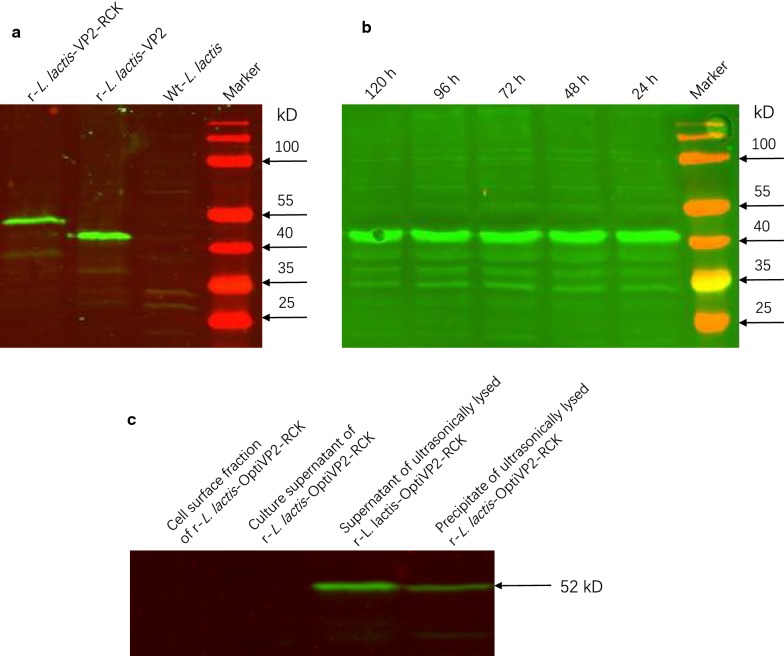
Identification of recombinant proteins and characterization of their stability via Western blotting analysis. **a** Immunoblot analysis of total whole-cell protein extracts from recombinant r-*L. lactis*-OptiVP2-RCK (lane 1), r-*L. lactis*-OptiVP2 (lane 2) and wt-*L. lactis* (lane 3). Proteins were separated on 12% SDS polyacrylamide gels and reacted with a VP2 Mab. **b** Detection of VP2-RCK fusion protein expression from recombinant r-*L. lactis*-OptiVP2 every 24 h for a total of 120 h via western blotting analysis (lanes 1–5). Sizes of the protein molecular mass standards are indicated to the left or right of each blot. **c** Western immunoblot analysis localization of VP2-RCK protein in recombinant r-*L. lactis*-OptiVP2-RCK cells. The cell surface fraction of r-*L. lactis*-OptiVP2-RCK (lane 1), culture supernatant of r-*L. lactis*-OptiVP2-RCK (lane 2), supernatant of ultrasonically lysed r-*L. lactis*-OptiVP2-RCK (lane 3), precipitate of ultrasonically lysed r-*L. lactis*-OptiVP2-RCK (lane 4). The sizes of molecular mass protein standards are indicated to the right

Expression of the recombinant protein VP2-RCK in r-*L. lactis*-OptiVP2-RCK NZ3900 was confirmed by western blotting using an anti-VP2 monoclonal antibody (Fig. 2a, c). The predicted molecular mass of a VP2-RCK specific band was observed at the expected size in the supernatant of ultrasonically lysed r-*L. lactis*-OptiVP2-RCK as well as in the precipitate of ultrasonically lysed r-*L. lactis*-OptiVP2-RCK (approximately 52 kDa, Fig. 2a, Lane 1; Fig. 2c, Lanes 3 and 4). Furthermore, a recombinant VP2 protein-specific band was observed at the expected size (approximately 47 kDa, Fig. 2a, Lane 2). This band was not observed in culture supernatants and cell surface extracts of r-*L. lactis*-OptiVP2-RCK or in wild-type (wt) *L. lactis* (Fig. 2c, Lanes 1 and 2; Fig. 2a, Lane 3). This finding suggests that the r-*L. lactis*-OptiVP2-RCK nisin-inducible expression system expresses the recombinant fusion protein VP2-RCK in a soluble form in the cytoplasm. Additionally, the western blotting results in Fig. 2b indicate that the recombinant protein VP2-RCK remained very stable in plasma at room temperature over the course of 5 days (Fig. 2b, Lanes 1–5).

**Fig. 3:**
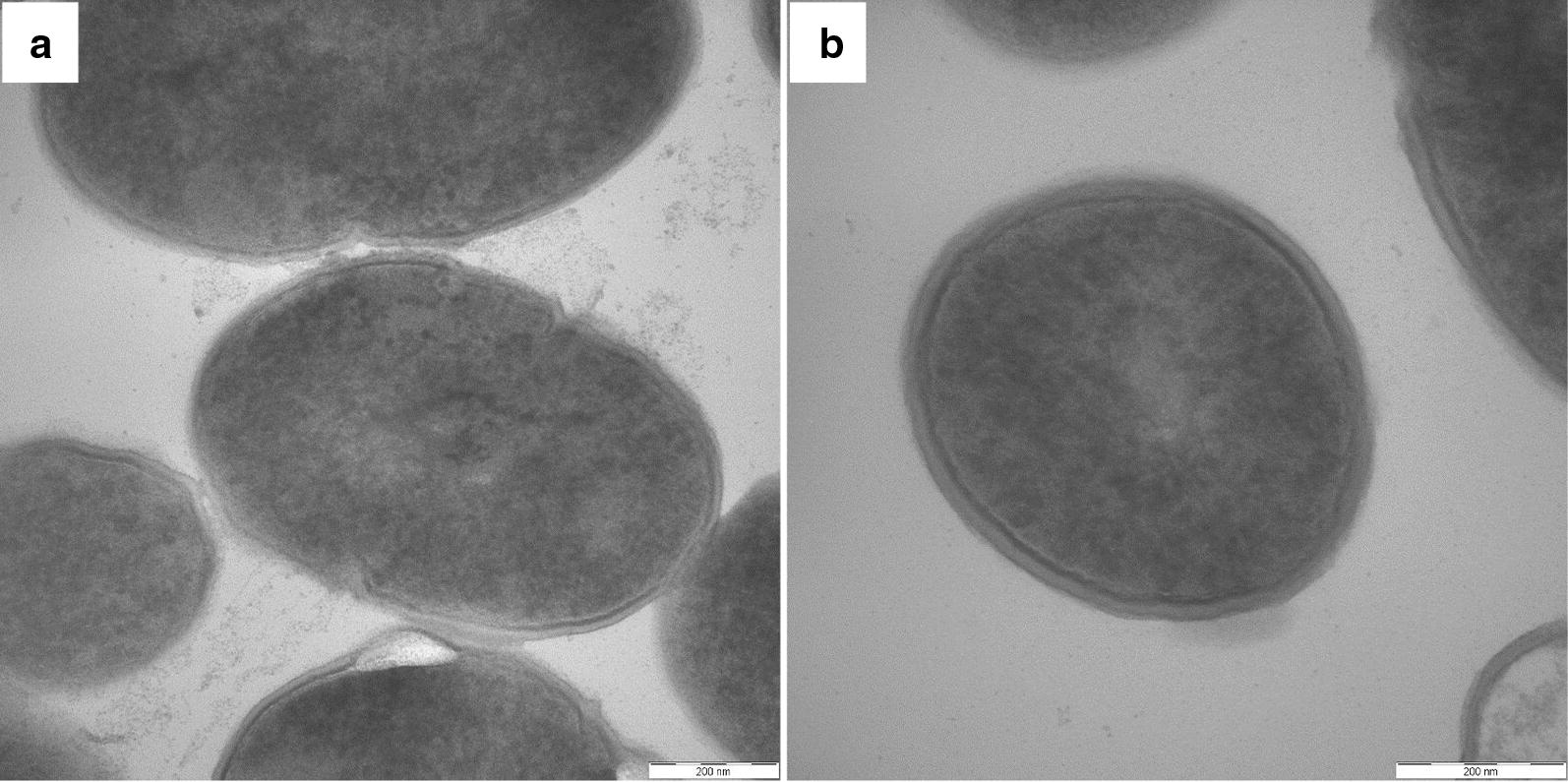
Ultrathin biopsy transmission electron microscopy analysis of recombinant LAB. **a** Recombinant r-*L. lactis*-OptiVP2-RCK cells were processed for ultrathin biopsy transmission electron microscopy analysis. No protein particles were observed in cell plasma. **b** The same process was performed for wt-*L. lactis*. No protein particles were observed (**a**, **b** bar = 200 nm)

Ultrathin biopsy transmission electron microscopy analysis showed that granule-like recombinant protein particles were not observed in the cell plasma, similar to wt-*L. lactis* (Fig. 3a, b). Thus, recombinant VP2-RCK protein does not polymerize to form particles but is soluble in the cytoplasm.

### Protection against lethal vvIBDV-HLJ0504 challenge

**Fig. 4:**
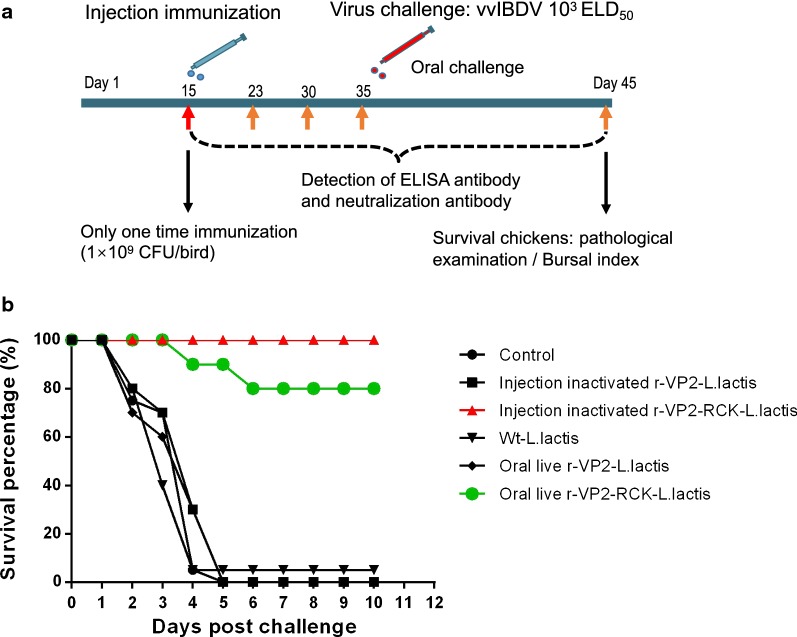
Animal experiment program and protective efficacy of recombinant r-*L. lactis*-OptiVP2-RCK against vvIBDV challenge. **a** Schematic diagrams of animal immunization, antibody detection and virus challenge. Briefly, SPF chickens were vaccinated at 15 days old. At 35 days old, chickens were challenged with 1000 ELD50 of vvIBDV viruses via intranasal and intraocular routes. And at 10 days post challenge (45 days old), surviving animals were sacrificed and necropsy was performed including pathological examination and bursal index. Throughout the animal experiment, serums were collected at 15, 23, 30, 35 and 45 days old for detect the ELISA antibody and neutralization antibody. **b** Protective efficacy of r-*L. lactis*-OptiVP2-RCK against vvIBDV (0504) challenge. Survival rates of chickens challenged with vvIBDV over an observation period of 10 days

Fifteen days old SPF chickens were vaccinated with 10^9^ CFU of recombinant r-*L. lactis*-OptiVP2-RCK NZ3900 per bird. At the age of 35 days, chickens were challenged with 10^3^ ELD50 (chicken embryo median lethal dose, ELD50) of vvIBDV virus via intranasal and intraocular routes. At 10 days post challenge (45 days old), surviving chickens were sacrificed, and necropsy was performed followed by pathological examination and bursal index measurement. Throughout the experiment, serum samples were collected at 15, 23, 30, 35, and 45 days of age to detect the ELISA antibody and neutralization antibody (Fig. 4a).

After 10 days of lethal vvIBDV-HLJ0504 challenge, chickens injected with inactivated r-*L. lactis*-OptiVP2-RCK demonstrated 100% full protection (15/15) with HBLS values less than 1. The oral live r-*L. lactis*-OptiVP2-RCK group demonstrated 80% protection (12/15) (Fig. 4b), and five HBLS values were 1 or less. In contrast, none of the chickens in the injected inactivated r-*L. lactis*-OptiVP2 group, the oral live r-*L. lactis*-OptiVP2 group or the control group (0/15) were protected against vvIBDV challenge (Fig. 4b).

**Fig. 5:**
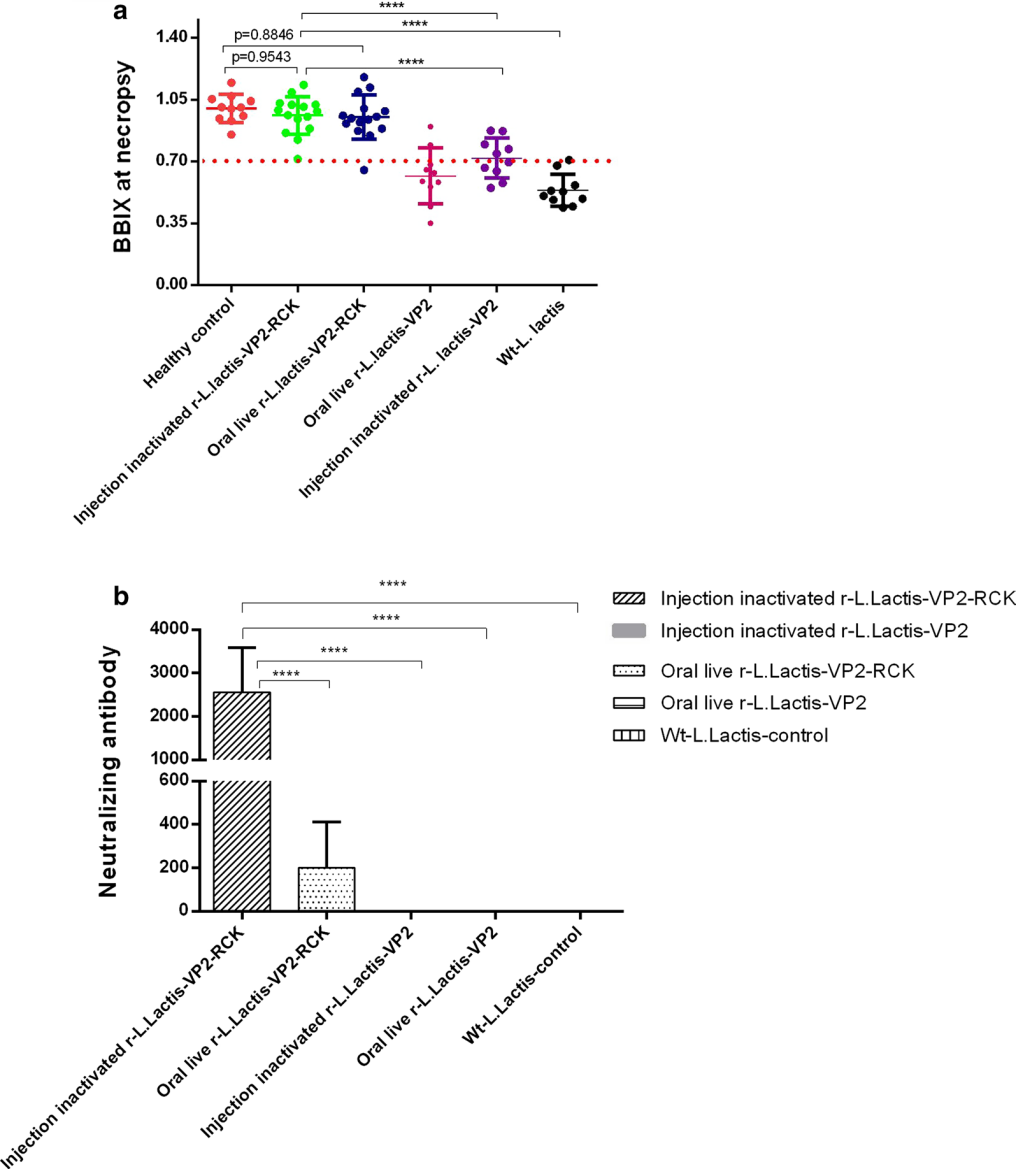
Analysis of protection against lethal vvIBDV-HLJ0504 challenge determined by calculating BBIX values and detecting specific neutralizing antibodies. **a** BBIX values of chickens after vvIBDV challenge. Dead birds were dissected on the day they died, and surviving birds were euthanized and analyzed after the observation period on day 10 post challenge. The data presented are the mean ± standard deviation (SD) from 15 birds in both the injected and oral r-*L. lactis*-OptiVP2-RCK groups, 11 birds in the healthy control group, and 10 birds in the oral r-*L. lactis*-OptiVP2 group. **b** Detection and comparison of virus neutralization antibodies against IBDV in chickens 14 days after vaccination. Statistical significance was set at *P < 0.05, **P < 0.01, ***P < 0.001, ****P < 0.0001

Histopathological examination revealed that the overall BBIX values of the injected inactivated r-*L. lactis*-OptiVP2-RCK group, the oral live r-*L. lactis*-OptiVP2-RCK group and the healthy control group were nearly all greater than 0.7 (Fig. 5a), while a third of chickens had BBIX scores greater than 0.7 in the oral live r-*L. lactis*-OptiVP2 group and the injected inactivated r-*L. lactis*-OptiVP2 group, and nearly all chickens had BBIX scores less than 0.7 in the wt-*L. lactis* group (Fig. 5a). Thus, r-*L. lactis*-OptiVP2-RCK confers efficient protection both via intramuscular and oral administration.

### r-*L. lactis*-OptiVP2-RCK immunization stimulates a VP2-specific immune response and only produces specific neutralizing antibodies

Serological ELISA antibody detection indicated that all serological ELISA antibody titers were negative, including in the control group. We did not detect any serum IgG responses against VP2 (data not shown). However, neutralization test results indicated high neutralizing antibody titers (approximately 1:2^10^ to 1: 2^12^) in the injected inactivated r-*L. lactis*-OptiVP2-RCK group and the oral live r-*L. lactis*-OptiVP2-RCK group after vaccination 2 weeks (Fig. 5b). The other three groups (oral live r-*L. lactis*-OptiVP2 group, injected inactivated r-*L. lactis*-OptiVP2 group and wt-*L. lactis* group) demonstrated no neutralizing antibodies (Fig. 5b). Based on these results, the novel inactivated recombinant r-*L. lactis*-OptiVP2-RCK stimulates the VP2-specific immune response and induces high levels of specific neutralizing antibodies that confer full protection against IBDV in chickens.

## Discussion

The *Lactococcus lactis* Serial derived strains (including *Lactococcus lactis* NZ3900 strain, original from *Lactococcus lactis MG1363*) are commercially employed as probiotics, with *Lactococcus lactis MG1363* being the model probiotic strain [19, 30, 31], *Lactococcus lactis* is industrially important microorganism used in many dairy fermentations as a homofermentative bacterium. Its functional characteristics that have extensively been studied in *Lactococcus* include the extracellular and intracellular proteolytic system, the carbon metabolism, the production of antibiotic substances, and their interaction with and resistance to bacteriophages. This wealth of knowledge and experience has led to the use of *Lactococcus* in several fields of biotechnology, e.g. the expression of bacterial and viral antigens for safe vaccination via mucosal immunization, the availability of an easy-to-operate and strictly controlled gene expression system (NICE^®^) has been crucial for the development of many of these applications [18, 32–36].

In this study, *Lactococcus lactis* NZ3900 (*lacF*−, *pepN: nisR nisK*) is a standard food-grade LAB strain that undergoes selection based upon its ability to grow on lactose. pNZ8149 is a broad host range vector containing *lacF*, which permits food-grade selection for growth on lactose, and the *nisA* promoter, which is followed by a *Nco*I site for translational fusions at the ATG. Together, *Lactococcus lactis* NZ3900 and the pNZ8149 vector constitute a tightly-controlled Nisin-regulated gene expression system (NICE^®^) developed by NIZO Food Research, NL [19, 37, 38]. This system is easy to operate and is advantageous for over-expressing homologous and heterologous genes for functional studies and for obtaining large quantities of specific gene products [18, 32–36].

Most pathogens colonize and invade the host at mucosal surfaces, such as the lung and the intestine. To combat intestinal pathogens the induction of local adaptive immune responses is required, which is mainly achieved through oral vaccination, in this study, the recombinant *Lactococcus lactis* (*L. lactis*) expressing the VP2 capsid antigen of IBDV within a newly characterized RCK-based “active” delivery system. RCK is an outer membrane protein encoded by the *rck* gene of *Salmonella enterica*. According to a previous study, RCK is necessary and sufficient to enable non-invasive *E. coli* and RCK-coated proteins to adhere to and invade different cells via a Zipper-like entry mechanism [29, 39]. Rosselin et al. confirmed that peptide 113-185 is involved in adhesion and internalization [29]. In this study, to further understand the role of the *rck* gene as an active delivery trigger, we extensively analyzed *rck* gene sequences deposited in the NCBI’s GenBank database (accession number LN974247) and optimized *rck* codons according to the reference genome of *Lactococcus lactis* (accession number NC_009004). We then sought to characterize the efficiency of a recombinant *L. lactis*-based inducible expression system containing the fusion antigen VP2-RCK and the immunogenicity of unadjuvanted oral and injected r-*L. lactis*-VP2-RCK in poultry. The main innovation in this paper suggest that the r-*L. lactis*-RCK system induces neutralizing antibodies, rendering it appropriate to use in mucosal vaccines to prevent and control IBDV infection in poultry.

In our study, r-*L. lactis*-VP2-RCK induced the production of a specific immune response characterized by neutralizing antibodies that provided full protection against vvIBDV challenge in chickens. Our results indicate that recombinant *L. lactis* delivers antigens effectively and efficiently. Additionally, only the expression of a VP2 and RCK fragment fusion produced this specific immune response against IBDV. This is the first evidence that the oral or injected administration of VP2 produced by LAB promotes a specific immune response mediated by neutralizing antibodies in chickens.

Unlike our study, previous work by Dieye et al. showed that recombinant *L. lactis* expressing an anchored Nuc-VP2 fusion induced a systemic, specific response against Nuc but not against VP2 [16]. Chatel et al. also showed that mice immunized with r-BLG produced by *Lactococcus* did not exhibit significant levels of BLG-specific IgA, IgG1, IgG2a, or IgE [26]. In contrast, our study detected high levels of serum neutralizing antibodies against IBDV in chickens. Although significant levels of specific neutralization antibodies were found in sera from chickens vaccinated both orally and by injection, both classical antibodies tested by ELISA were absent. There are many possible explanations for this observation. Based on observations in mice [40], differences in serum neutralizing antibodies reflect different mucosal and systemic immune responses. Additionally, differences in the VP2-RCK fusion protein, such as its topology in the cytoplasm, may account for the differing results. Specifically, the fusion protein may develop a new spatial structure, necessitating a new processing and delivery pathway to display the VP2 moiety to immune cells in the form of a different epitope. In order to prove VP2-specific response authentically, we conducted western blot analysis using the polyclonal serum antibodies obtained from the vaccinated bird after 2 weeks of vaccination to detect the recombinant protein VP2-RCK in r-*L. lactis*-OptiVP2-RCK NZ3900. The protein band of the molecular mass equivalent to that of VP2-RCK band was clearly observed (data not shown) indicating the effectiveness of the vaccine. Such a scenario could possibly explain why a neutralizing antibody response against VP2 was observed. A normal ELISA assay would not detect these antibodies. However, these hypotheses must be tested, for example, whether antigens from recombinant *L. lactis* expressing a foreign VP2-RCK protein are delivered to DCs by a particular pathway, whether neutralizing antibodies produced in the bird simply block only the replicating viruses (against the replicating VP2 protein), and so on. However, although only one vaccination could provide full protection against IBDV challenge in chickens, there are still a series of questions need to be answered. For example, whether this mechanism is only applicable to chickens, and whether the specific antigen expression and delivery system can be applicable to induce the production of high neutralizing antibodies in other animals. Therefore, further studies must be carried out to ascertain whether our results are applicable to other antigens and whether it is possible to induce similar specific responses.

In summary, recombinant *L. lactis* expressing a foreign protein constitutes a unique antigen-expression and specific-delivery system to produce neutralizing antibodies and is a powerful tool to develop a new vaccine carrier. In the emerging field of antigen delivery by LAB, further work is needed to better understand and improve the delivery mechanisms that induce specific immune responses accompanied by neutralizing antibodies. In future studies, it may be possible to improve this specific immune response in two ways: first, by anchoring antigens to the cell wall, and second, by co-expressing antigens and adjuvants. Such improvements may render LAB an ideal oral live vector vaccine.

## Conclusion

In this work, we geared to produce a new vaccine against infectious bursal disease virus, here, we construct a recombinant lactic acid bacterium that produce the fusion protein with the RCK protein of *Salmonella enterica* and the VP2 of infectious bursal disease virus. We find recombinant *L. lactis* (r-*L. lactis*-OptiVP2-RCK) was able to produce a soluble and stable form of VP2-RCK in the cytoplasm. Animal experiments show that recombinant lactic acid bacteria could induce high levels of neutralizing antibodies and provide full protection against IBDV challenge in chickens, which suggest that *L. lactis* is a potential tool for developing vaccines. But the serum antibody ELISA titers were negative after immunization, which remind that the recombinant lactic acid bacteria active delivery system in this work is different from other routine IBDV vaccines. The primary mechanism that produces immune protection need further research.

## Methods

### Bacterial strains, media and growth conditions

The bacterial strains and plasmids used in this study are listed in Table 1. In the LAB strain *L. lactis* NZ3900, the *nisK* and *nisR* genes were inserted into the *pepN* gene site, the lactose operon was integrated into the chromosome, and the *lacF* gene was deleted. Deletion of the *lacF* gene inhibits the growth of this strain on lactose unless *lacF* is present on a plasmid [41]. We used the plasmid pNZ8149 (MoBiTec, Goettingen, Germany).

**Table 1:** Bacterial strains and plasmids used in this study

Strain or plasmid	Relevant characteristics	Source/reference
Strains		
*NZ3900*	Host for food-grade use of the NICE system by plasmids election based on the ability to grow on lactose as carbon source.	MoBiTec
*L. lactis*	*L. lactis NZ3900* containing *pNZ8149* plasmid	MoBiTec
*L. lactis*-*RCK*	*L. lactis NZ3900* containing *pNZ8149*-*RCK* plasmid	This study
*L. lactis*-*VP2-RCK*	*L. lactis NZ3900* containing *pNZ8149*-*VP2*-*RCK*	This study
Plasmid		
*pNZ8149*	High-copy number lactococcal vector	MoBiTec
*pNZ8149*-*RCK*	Vector carrying rck gene	This study
*pNZ8149*-*VP2*-*RCK*	Vector carrying VP2 gene and rck gene	This study

*Lactococcus lactis* NZ3900 competent cells were grown in GEM medium (0.5% glucose Elliker medium) at 30 °C without agitation, adding 0.5% lactose (as a sole carbon source) as necessary.

### DNA manipulation and recombinant plasmid construction

The opti-*rck* gene of *Salmonella enterica* subsp. *enterica* was amplified using the forward primer 5′-TGGGTACTGCAGGCATGCTTGGTACCGGACGTGCAGAAGT-3′ and the reverse primer 5′-TCTCTAGAACTAGTGGTACCTTACTAGAGAACAACATTTT-3′ and cloned into the *L. lactis*-based expression plasmid pNZ8149 (MoBiTec, Goettingen Germany) by homologous recombination and the One-Step Cloning kit (Vazyme Biotech Co., Nanjing, China). The resulting plasmid, pNZ8149-RCK, was linearized with *Nco*I and *Sph*I. The Opti-VP2 gene was also amplified with the forward primer 5′-ATTATAAGGAGGCACTCACCATGGCTAATTTACAAGATCA-3′ and the reverse primer 5′-ACTTCTGCACGTCCGGTACCTGCTCCAGCAATTTTCAATG-3′ from the plasmid pUC57-Opti-VP2 (kindly codon-optimized and synthesized by Nanjing GenScript Biotechnology Corporation, China), and cloned into the plasmid pNZ8149-RCK by homologous recombination and the One-Step Cloning kit (Vazyme Biotech Co., Nanjing, China). The identity of the resulting plasmid pNZ8149-OptiVP2-RCK was confirmed by *Nco*I and *Kpn*I digestion (Fig. 1a) (*Geneious version 10.2 created by Biomatters.* Available from https://www.geneious.com). Cloned regions were sequenced after each stage of construction, and the final recombinant plasmid was electro-transformed into competent *L. lactis* NZ3900. The selected positive clone was named r-*L. lactis*-OptiVP2-RCK. Recombinant strains were grown in GEM at 30 °C without shaking.

### Nisin-controlled expression, cell fractionation and protein extraction

Nisin-controlled expression was carried out as previously described [42, 43] Briefly, overnight cultures of *L. lactis* NZ3900 harboring the pNZ8149-OptiVP2-RCK plasmid were inoculated in 5 mL of fresh Elliker-medium (EM) containing 0.5% lactose and incubated at 30 °C without shaking. The next day, the cultures were diluted 1/25 in 2 tubes 15-mL culture containing fresh medium and grown until the optical density at 600 nm (OD600) reached values between 0.4 and 0.5. At this point, nisin was added to a final concentration of 10 ng/mL in a 15-mL culture, and the other 15-mL culture served as a negative control. To induce nisin-controlled expression, cultures were incubated for 2 to 3 h. Then, cells were collected, and the medium was tested for protein production.

Recombinant protein extraction. Protein extraction was performed on medium, supernatants obtained from ultrasonic lysis, cell wall fractions, and protoplast fractions as previously described [44]. Briefly, 10 mL of an exponential-phase culture (OD600 of 0.6 to 0.8) was microcentrifuged at 4 °C for 3 min at 15,000×*g*. The supernatant and the cell pellet were processed separately. The supernatant was filtered through 0.45-μm pore-size filters (low protein retention; Millipore, USA) to remove bacteria, and the filtered supernatant was then added to an Amicon^®^ Ultra-15 30 kDa Centrifugal Filter Device (Millipore). The device was centrifuged at a maximum of 5000×*g* for approximately 40–60 min using a swinging bucket rotor, and the collected tenfold concentrated supernatant was used for Western blotting. Cell wall fractions were prepared for further analysis as previously described [44, 45], Briefly, For cell wall preparations, Cell pellet of 10 mL culture were resuspended in 1 mL of 10 mM Tris–Cl (pH 8.0) containing 30% raffinose, 100 U of mutanolysin per mL, 1 mg of lysozyme per ml, and complete protease inhibitor (GE). Cells were digested for 3 h at 37 °C with constant rotation and pelleted by centrifugation, the supernatant containing the cell wall fraction was used for further analysis. The remaining cell pellets were lysed by ultrasonication for further protein analysis.

### Western blotting analysis, identification of recombinant proteins and in vitro testing of recombinant protein stability

For immunoblot analysis, all prepared protein samples were separated by 8–12% SDS-PAGE and electroblotted onto a nitrocellulose membrane (Bio-Rad) for detection using a VP2 monoclonal antibody at a 1:2000 dilution [12].

Ultrathin biopsy transmission electron microscopy was performed to analyze recombinant LAB. r-*L. lactis*-OptiVP2-RCK was grown as described above, harvested by centrifugation and washed with phosphate-buffered saline (PBS). The centrifuged pellet was used to generate ultrathin sections, and transmission electron microscopy was performed to observe whether granule-like recombinant protein particles were present in cell plasma. Cultured *Lactococcus lactis NZ3900* expressing recombinant protein was placed at room temperature, and each day an aliquot of recombinant *L. lactis*-OptiVP2-RCK was taken for Western blot analysis to observe whether the recombinant proteins are stable in the bacteria.

### Preparation of *r*-*L. lactis* for immunization and protection against vvIBDV challenge

All recombinant strains of *L. lactis* NZ3900 (harboring the corresponding plasmid) were inoculated and induced with nisin as described above. The collected bacterial pellets were washed two times with sterile PBS, the final pellets were suspended in sterile PBS supplemented with 0.5% lactose for immunization at an appropriate concentration (1 × 10^9^ CFU/mL recombinant *L. lactis* NZ3900 and controls in a 500 μL volume), and then inactivated at 70 °C for 10 min.

To evaluate the protective efficacy of recombinant r-*L. lactis*-OptiVP2-RCK NZ3900 against vvIBDV (HLJ0504), all chickens were maintained under specific-pathogen-free (SPF) negative pressure isolator conditions for all experiments. According to the experimental schematic in Fig. 4a, three groups were immunized via intramuscular administration of 500 μL of wt-*L. lactis*, 500 μL of inactivated r-*L. lactis*-OptiVP2-RCK or 500 μL of inactivated r-*L. lactis*-OptiVP2. Two groups were immunized orally with 500 μL of live r-*L. lactis*-OptiVP2-RCK or 500 μL of live r-*L. lactis*-OptiVP2. In addition, one group was a non-immune healthy control group. All chickens were challenged with 10^3^ ELD50 of vvIBDV (HLJ0504) at 35 days post vaccination as previously described [12, 46, 47].

### Clinical observation and histopathological examination

Dead and surviving chickens were evaluated for bursal atrophy by determining the bursa:body-weight index (BBIX) as previously described [1, 48, 49]. Chicken tissue samples of the bursa of Fabricius were immediately placed into 10% neutral-buffered formalin for histopathological examination. Bursal lesion scores (HBLS) were recorded by performing histopathological examination as previously described [1, 2, 10, 50]. Briefly, a microscopic HBLS score was determined on the following scale of 0 to 5.0, no abnormalities; (1) 1–20%; (2) 21–40%; (3) 41–60%; (4) 61–80%; and (5) 81–100% lymphocyte depletion. An HBLS value of no more than 1 (no or slight lesion) was defined as protective against IBDV challenge [1].

### Serological ELISA antibody detection and neutralization test

Weekly serum samples collected after immunization were tested by performing VP2-coated ELISA (IDEXX IBD-XR Ab Tests kit; IDEXX, Westbrook, Maine, USA) and whole virus-coated ELISA (IDEXX IBD Ab Tests kit) following the manufacturer’s direction. A virus neutralization (VN) assay was performed as previously described [46, 47, 51]. Briefly, serum samples collected in triplicate were serially diluted two-fold and mixed with equal volumes of the cell culture-adapted HLJ0504 virus at 100 TCID50. After 60 min of incubation at 37 °C, the mixtures were added to 100% confluent CEFs, followed by further incubation for 3 days. VN titers were designated as the highest dilutions at which there were no visible cytopathic effects.

### Statistical analysis of data

Statistical analyses were carried out using ordinary one-way ANOVA to evaluate statistical differences among groups, and P values of 0.05 were considered statistically significant. P < 0.01 was considered highly significant, P < 0.001 was considered very highly significant and P < 0.0001 was considered extremely highly significant.

### Ethics statement

All animal experiments, such as generation of antisera from SPF chickens and immune protection tests of commercial vaccines, were carried out according to protocols approved by the ethical review board of Harbin Veterinary Research Institute (HVRI) of the Chinese Academy of Agricultural Sciences (CAAS) and performed in accordance with approved animal care guidelines and protocols. The animal ethics committee approval number is SYXK (Heilongjiang) 2011022.
